# Investigation of selected genomic deletions and duplications in a cohort of 338 patients presenting with syndromic obesity by multiplex ligation-dependent probe amplification using synthetic probes

**DOI:** 10.1186/s13039-014-0075-6

**Published:** 2014-10-31

**Authors:** Carla S D’Angelo, Monica C Varela, Cláudia IE de Castro, Chong A Kim, Débora R Bertola, Charles M Lourenço, Ana Beatriz A Perez, Celia P Koiffmann

**Affiliations:** Human Genome and Stem Cell Center, Department of Genetics and Evolutionary Biology, Institute of Biosciences, University of Sao Paulo, Sao Paulo, Brazil; Genetics Unit, Department of Pediatrics, Children Institute, School of Medicine, University of Sao Paulo, Sao Paulo, Brazil; Neurogenetics Unit, Department of Medical Genetics, School of Medicine, University of Sao Paulo, Ribeirao Preto, Brazil; Department of Morphology, Medical Genetics Center, Federal University of Sao Paulo, Sao Paulo, Brazil

**Keywords:** Obesity, Developmental delay, Copy number variants (CNVs), Multiplex ligation-dependent probe amplification (MLPA), Chromosomal microarray analysis (CMA)

## Abstract

**Background:**

Certain rare syndromes with developmental delay or intellectual disability caused by genomic copy number variants (CNVs), either deletions or duplications, are associated with higher rates of obesity. Current strategies to diagnose these syndromes typically rely on phenotype-driven investigation. However, the strong phenotypic overlap between syndromic forms of obesity poses challenges to accurate diagnosis, and many different individual cytogenetic and molecular approaches may be required. Multiplex ligation-dependent probe amplification (MLPA) enables the simultaneous analysis of multiple targeted loci in a single test, and serves as an important screening tool for large cohorts of patients in whom deletions and duplications involving specific loci are suspected. Our aim was to design a synthetic probe set for MLPA analysis to investigate in a cohort of 338 patients with syndromic obesity deletions and duplications in genomic regions that can cause this phenotype.

**Results:**

We identified 18 patients harboring copy number imbalances; 18 deletions and 5 duplications. The alterations in ten patients were delineated by chromosomal microarrays, and in the remaining cases by additional MLPA probes incorporated into commercial kits. Nine patients showed deletions in regions of known microdeletion syndromes with obesity as a clinical feature: in 2q37 (4 cases), 9q34 (1 case) and 17p11.2 (4 cases). Four patients harbored CNVs in the DiGeorge syndrome locus at 22q11.2. Two other patients had deletions within the 22q11.2 ‘distal’ locus associated with a variable clinical phenotype and obesity in some individuals. The other three patients had a recurrent CNV of one of three susceptibility loci: at 1q21.1 ‘distal’, 16p11.2 ‘distal’, and 16p11.2 ‘proximal’.

**Conclusions:**

Our study demonstrates the utility of an MLPA-based first line screening test to the evaluation of obese patients presenting with syndromic features. The overall detection rate with the synthetic MLPA probe set was about 5.3% (18 out of 338). Our experience leads us to suggest that MLPA could serve as an effective alternative first line screening test to chromosomal microarrays for diagnosis of syndromic obesity, allowing for a number of loci (e.g., 1p36, 2p25, 2q37, 6q16, 9q34, 11p14, 16p11.2, 17p11.2), known to be clinically relevant for this patient population, to be interrogated simultaneously.

## Background

Obesity is defined as an abnormal or excessive fat accumulation that presents a risk to health, and is commonly classified using the body mass index (BMI = weight/height2). It is well established that single-gene defects or genomic copy number variants (CNVs), either deletions or duplications, can lead to both syndromic and non-syndromic forms of obesity [1]. The term syndromic obesity refers to rare or uncommon genetic syndromes, in which obesity is one of a range of symptoms, often including developmental delay (DD) and intellectual deficit (ID). Prader-Willi syndrome (PWS; OMIM 176270) is the most common known genetic cause of obesity, resulting more frequently from a deletion in the paternal 15q11.2-q13 chromosomal region. The diagnosis is typically made in early infancy due to hypotonia and poor feeding, prior to the onset of obesity and hyperphagia [2].

Several other clinically well-defined microdeletion syndromes also have an increased prevalence of obesity, but they are often clinically difficult to diagnose due to extensive phenotypic overlap and lack of a diagnostic testing path. Examples are 1p36 monosomy (OMIM 607872), brachydactyly-mental retardation (BDMR) syndrome (OMIM 600430), 6q16 deletion syndrome (OMIM 603128), Kleefstra syndrome (KS; OMIM 610253), Wilms tumor, aniridia, genitourinary anomalies, and mental retardation (WAGR) syndrome (OMIM 194072, 612469), and Smith-Magenis syndrome (SMS; OMIM 182290). Some of these syndromes have now been explained by haploinsufficiency of a single gene in the critical deletion intervals, including BDMR (*HDAC4*) [3], KS (*EHMT1*) [4], and SMS (*RAI1*) [3-5]. In addition, the obesity phenotype in patients with 6q16 deletion is likely explained by haploinsufficiency of the *transcription factor single-minded 1* (*SIM1*) gene [6], whereas obesity susceptibility in WAGR syndrome mainly depends on haploinsufficiency for the *brain-derived neurotrophic factor* (*BDNF*) gene [7].

Since in recent years chromosomal microarray analysis (CMA) has become the method of choice for detecting copy number imbalances in the genome, a great number of rare CNVs and microdeletion/duplication syndromes have been found that cause, or predispose to, obesity along with DD/ID and other phenotypic findings, such as 1p21.3 microdeletions [8], 2p25.3 deletions [9,10], interstitial deletions within 6q14.1q15 [11], 6q22 deletions [12], translocation der(8)t(8;12)(p23.1;p13.31) [13], interstitial deletions on 11p14.1 [14], 12q subtelomeric deletions [15], 16p11.2 distal and proximal deletion [16,17], 17q24.2 microdeletions [18], chromosome 19q duplications [19], and several others [20-22]. Multiplex ligation-dependent probe amplification (MLPA) is more cost and time-efficient than microarray-based approaches, and provides an alternate method of simultaneously determining copy number status at multiple target loci. In this study, we have designed a synthetic probe set to interrogate the copy number status at previously described loci associated with syndromic obesity, and defined the length of the lost or gained DNA regions by SNP-array and/or oligoarray-CGH or, alternatively, using commercial MLPA kits. We report our findings from screening in a cohort of 338 patients with syndromic obesity.

## Results

Results are shown in Table 1. Copy number imbalances were detected in 18 patients (diagnostic yield of 5.3%); 18 deletions and 5 duplications. The alterations in ten cases were confirmed (delineated) by SNP-array (patients 2 and 18) and oligoarray-CGH (patients 1, 3–8, 17), while the alterations in eight cases were fine-mapped using the commercial MLPA kits P064-B2 (patients 9–12) and P023-B (patients 13–16), with more probes in sequences adjacent to the genes in which copy number gains or losses were detected.

**Table 1:** **Chromosomal aberrations detected primarily by the MLPA testing panels**

**Case**	**Cytoband**	**CNV type**	**Aberrant MLPA probe(s)**	**CMA/ MLPA**	**Genomic coordinates**	**Size (Mb)**	**Inheritance**	**Phenotype**
P1	1q21.1	Gain	PRKAB2, ACP6	180 K oligoarray	chr1:146.07-147.83 Mb	1.8	n.d.	6.8 yr old male (BMI >95^th^), speech delay (>2 yr), macrocephaly (>98^th^), accelerated growth (90-95^th^), genital hypoplasia
P2	2q37.1q37.2	Gain	--	500 K SNP array	chr2:235.09-236.8 Mb	1.7	--	11 yr old male (BMI >95^th^), DD (walked: 3.6 yr, spoke: 3 yr); ID, hypotonia, motor and speech impairment, hyperphagia, seizures, macrocephaly (98^th^), facial dysmorphisms, inverted nipples, unilateral cryptorchidism
	2q37.2q37.3	Loss	HDAC4, GPR35		chr2:236.94-243.01 Mb	6.1	de novo	
P3	2q37.2q37.3	Loss	HDAC4, GPR35	60 K oligoarray	chr2:236.85-243.0 Mb	6.2	n.d.	21 yr old male; DD, hypotonia, hyperphagia, obesity, absent speech, mild dysmorphisms, supernumerary teeth, unilateral cryptorchidism, micropenis
P4	2q37.2q37.3	Loss	HDAC4, GPR35	60 K oligoarray	chr2:237.22-243.0 Mb	5.8	de novo	8 yr old female (BMI >95^th^), DD (walked: 17mo, spoke: >2 yr), learning disability, dolichocephaly, facial dysmorphisms, mamilar hypertelorism, inverted nipples, brachydactyly, 2–3 toe syndactyly, hirsutism, joint hypermobility
P5	2q37.3	Loss	GPR35	180 K oligoarray	chr2:240.88-243.03 Mb	2.2	n.d.	5 yr old female (BMI >95^th^), DD (walked: 2 yr, spoke: 3 yr), ID, behavior problems, prominent ear, thin elongated eyebrow, strabismus
	17q25.3	Gain	--		chr17:78.77-81.06 Mb	2.3		
P6	9q34.3	Loss	EHMT1	60 K oligoarray	chr9:140.67-141.02 Mb	0.4	de novo	9.5 yr old female (BMI >95^th^), DD (walked: 18mo, spoke: 6 yr), hypotonia, hyperphagia, behavior problems, tall stature (>97^th^)
P7	16p11.2	Loss	SH2B1	180 K and 60 K oligoarray	chr16:28.82-29.04 Mb	0.2	de novo	7 yr old female (BMI >95^th^), GDD, hypotonia, ADHD, hyperphagia, speech impairment, deep-set eyes, straight eyebrows, thick earlobe
P8	16p11.2	Gain	CDIPT, MAPK3	180 K and 60 K oligoarray	chr16:29.65-30.19 Mb	0.5	paternal	8.5 yr old male, GDD, hyperphagia, obesity [sic], speech impairment, behavior problems, scoliosis
P9	17p11.2	Loss	FLCN,^1^ RAI1	P064 kit	chr17:17.13-19.29 Mb	2.2	de novo	11 yr old male (BMI >95^th^), DD, ID, hyperphagia, behavior and sleep problems, macrocephaly (>98^th^), typical facial dysmorphisms, brachydactyly, 2–3 toe syndactyly, micropenis
P10	17p11.2	Loss	FLCN,^1^ RAI1	P064 kit	chr17:16.85-19.29 Mb	2.4	de novo	10 yr old male (BMI >95^th^), DD, ID, hypotonia, hyperphagia, behavior and sleep problems, speech and hearing impairment, facial dysmorphism
P11	17p11.2	Loss	FLCN,^1^ RAI1	P064 kit	chr17:16.85-19.29 Mb	2.4	de novo	7 yr old female (BMI >95^th^), DD (walked: 2 yr, spoke: 5 yr), ID, behavior problems, myopia, strabismus, astigmatisms, hypoplasia genital, 2–3 toe syndactyly
P12	17p11.2	Loss	FLCN,^1^ RAI1	P064 kit	chr17:16.85-19.29 Mb	2.4	n.d.	6.8 yr old male (BMI >95^th^), DD (walked: 5 yr, spoke: >4 yr), hypotonia, hyperphagia, compulsive behavior, typical facial dysmorphisms
P13	22q11.21	Loss	CRKL	P023 kit	chr22:19.32-21.35 Mb	2.0	de novo	3 yr old male (weight >97^th^), GDD, absent speech, hypotonia, mild facial dysmorphism
P14	22q11.21	Loss	CRKL	P023 kit	chr22:19.32-21.35 Mb	2.0	de novo	9 yr old male (BMI >95^th^), ID, hypotonia, speech delay (>3 yr), behavior problems, ADHD, hyperphagia, accelerated growth (90-95^th^), hypogonadism
P15	22q11.21	Loss	CRKL	P023 kit	chr22:19.32-21.35 Mb	2.0	paternal	13mo old male (BMI 76^th^), DD, hypotonia, growth delay (3^rd^-5^th^), relative overweight (weight to height ratio >75th percentile), dolichocephaly, facial dysmorphisms
P16	22q11.21	Gain	CRKL	P023 kit	chr22:19.32-21.35 Mb	2.0	paternal	8.6 yr old male (weight >97^th^), DD (walked: >2 yr), mild ID, hypotonia, facial dysmorphisms, widened mediastinum, brachydactyly, micropenis
P17	22q11.22q11.23	Loss	RAB36	180 K oligoarray	chr22:23.01-23.65 Mb	0.6	maternal	2.8 yr old female (BMI 85^th^), hypotonia, non-ambulatory and non-verbal, epilepsy (onset at 3mo), brachycephaly, deep-set eyes, visual impairment, joint laxity
P18	22q11.22q11.23	Loss	RAB36	500 K SNP array	chr22:23.06-23.7 Mb	0.6	n.d.^2^	15 yr old male (BMI >95^th^), GDD, ID, hypotonia, absent speech, behavioral and sleep problems, facial dysmorphisms, strabismus, hyperprolactinemia, micropenis

Breakpoints based on the coordinates of the first and last altered array probes. The alterations of patients 9–12 and 13–16 were fine-mapped by additional MLPA probes in the commercial kits P064-B2 and P023-B, respectively. Probes in the MLPA kit P064-B2 covering the 17p11.2 region are TNFRSF13B, LRRC48, LLGL1, PRPSAP2 and MFAP4. Probes in the MLPA kit P023-B covering the 22q11 region are IL17R, BID, HIRA, CLDN5, KIAA1652, KLHL22, PCQAP, SNAP29, LZTR1 and MIF.

^1^The FLCN probe is included in the MLPA P200 and P300 reference kits. It is located within the SMS region on chromosome 17p11.2, and was found deleted in all patients with deletions in RAI1. ^2^Adopted child. Not determined (n.d.); years (yr); months (mo). ID, intellectual disability; DD, developmental delay; GDD, global DD; ADHD, attention-deficit hyperactivity disorder.

**Figure 1:**
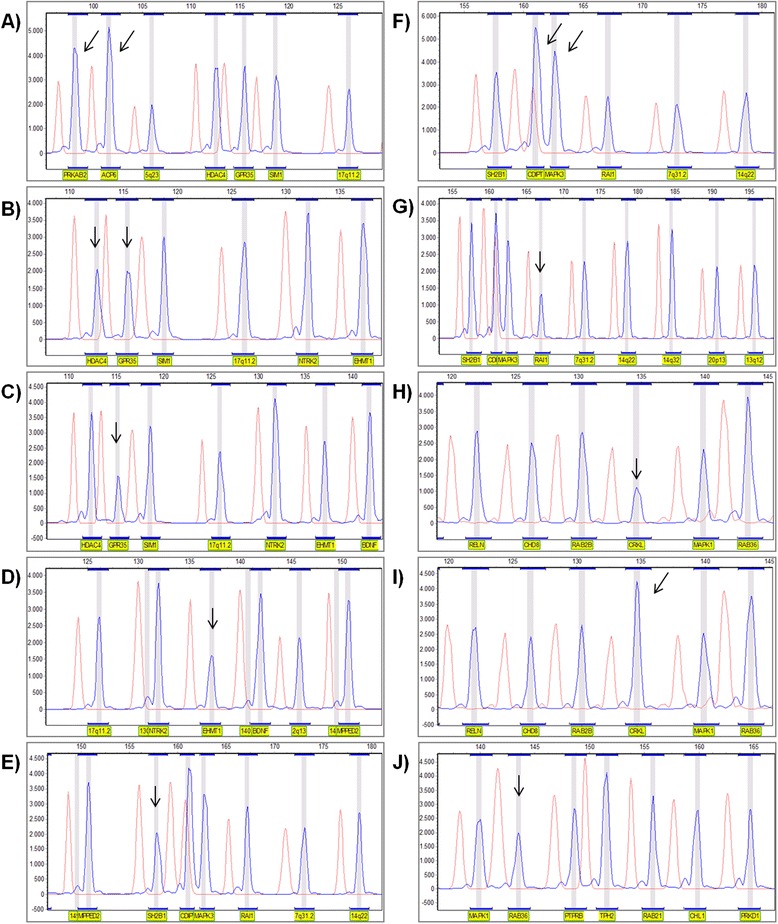
**Partial electropherograms for control individuals (red) and for the patient (blue) normalized by the GeneMarker software showing the custom probes with reduced or amplified peak heights (arrows). A)** sample with increased copy number for the probes PRKAB2 and ACP6. **B)** sample with reduced copy number for the probes HDAC4 and GPR35. **C)** sample with reduced copy number for the probe GPR35. **D)** sample with reduced copy number for the probe EHMT1. **E)** sample with reduced copy number for the probe SH2B1. **F)** sample with increased copy number for the probes CDIPT and MAPK3. **G)** sample with reduced copy number for the probe RAI1. **H)** sample with reduced copy number for the probe CRKL. **I)** sample with increased copy number for the probe CRKL. **J)** sample with reduced copy number for the probe RAB36.

**Figure 2:**
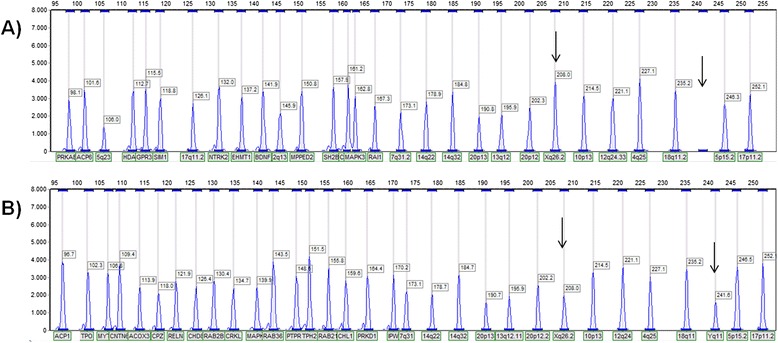
**Electropherograms patterns using control DNAs.** The two MLPA testing panels are shown (‘**A**’ probe set added to P300; ‘**B**’ probe set added to P200). Each peak represents the amplicon signal from a correspondent gene or control probe loci as labeled at bottom X-axis. The top X-axis indicates the size of the amplicon and the Y-axis indicates the fluorescent intensity. Arrows indicate X and Y chromosome specific control probes. For normal female control **(A)**, it was observed absence of Y-specific DNA sequences. For normal male sample **(B)**, it was observed a decrease for the dosage of X-specific control probe with respect to a female control.

Patient 1 had duplication for two gene probes from 1q21.1, PRKAB2 and ACP6 (Figure 1A). The duplication was shown to have a size of ~1.8 Mb with breakpoints in segmental duplication (SD), or low-copy repeat (LCR), blocks BP3 (distally) and BP4 (proximally), located in the distal 1q21.1 region (as delimitated by CMA). Four patients were deleted for probes mapped to the 2q37.3 locus: patients 2–4 had deletion for probes HDAC4 and GPR35 (Figure 1B), and patient 5 had a deletion for probe GPR35 (Figure 1C); no deletion of the region detected by probe HDAC4 was observed in the latter case. The CMA results showed an additional ~1.7 Mb duplication adjacent and proximal to the deletion of patient 2, and a concurrent 17q25.3 duplication of ~2.3 Mb in patient 5. Present deletions vary in size from ~2.2 Mb to 6.2 Mb. Patient 6 had a deletion for the 9q34.3 probe EHMT1 (Figure 1D). Subsequent CMA demonstrated a submicroscopic deletion of ~0.4 Mb extending from *EHMT1* (exon 17) to the most distal gene *CACNA1B*. Two patients were found with CNVs at the 16p11.2 locus: patient 7 had a deletion for the most distal probe SH2B1 (Figure 2E), and patient 8 had duplication for the most proximal probes CDIPT and MAPK3 (Figure 2F). Testing of parental DNAs showed that the deletion occurred apparently *de novo*, while the duplication was inherited from the normal father. CMA confirmed that the deletion in patient 7 is confined to the distal 16p11.2 region flanked by SD blocks BP2 and BP3, and the duplication in patient 8 to the proximal 16p11.2 region flanked by SD blocks BP4 and BP5.

Deletion in 17p11.2 probe RAI1 has been found in patients 9–12 (Figure 2G), and in all of these patients deletion within the *FLCN* gene, which is reference probe in the P200 and P300 MLPA kits, mapped to chromosome 17p11.2, has been detected (data not shown). The P064-B2 MLPA kit used in this study includes five probes specific for sequences in the SMS 17p11.2 region, with the most distal probe in the gene *TNFRSF13B*, near the distal SMS repeat (SMS-REP), and the most proximal probe in *MFAP4,* between the middle and proximal SMS-REPs. According to these MLPA results, the deletion of patient 9 has a minimum size of 2.2 Mb, with the distal breakpoint occurring between probes TNFRSF13B (not deleted) and FLCN (deleted), and extended proximally through MFAP4 (deleted). The deletions of patients 10–12 have a minimum size of 2.4 Mb mapping between deleted MLPA probes TNFRSF13B and MFAP4.

The remaining 6 patients had CNVs at the 22q11.2 locus: patients 13–15 had a deletion for the most proximal probe CRKL (Figure 2H), patient 16 had a duplication for this same probe (Figure 2I), and patients 17 and 18 had a deletion for the most distal probe RAB36 (Figure 2J); no deletion of the region detected by probe MAPK1 was observed in any of these patients. Two of the CNVs were apparently *de novo* (patients 13 and 14), while three CNVs were inherited from an unaffected parent (patients 15–17); the inheritance could not be explored for patient 18 who is an adopted teen. According to the results of the P023-B MLPA probes, all deletions and the duplication of proximal 22q11.2 have a minimum size of 2 Mb mapping between deleted MLPA probes HIRA proximally, which is located between LCR22-A and LCR22-B, and LZTR1 distally, located between LCR22-C and LCR22-D. The deletions of patients 17 and 18 were delineated by CMA and shown to have a size of ~0.6 Mb. The deletions boundaries are within SD blocks LCR22-E and LCR22-F at the distal 22q11.2 region.

## Discussion

In the present study, we aimed to develop an alternative, more cost-efficient tool than chromosomal microarrays to be used in the initial screening of a large cohort of patients presenting with syndromic obesity. As many of the currently known loci associated with syndromic forms of obesity have their causative genes already discovered, or have their breakpoints localized to flanking repeat sequences, we thought these disorders would be amenable to detection by MLPA using a synthetic probe set. In our cohort of 338 patients, 5.3% (18 patients) had a pathogenic diagnosis detected primarily by MLPA. Our detection rate is lower compared with 22% of patients with a potentially pathogenic diagnosis reported by Vuillaume *et al*. [22] for array-CGH in a similar cohort. The lesser diagnostic yield in the present study may be explained by the targeted approach versus the whole genome approach in Vuillaume’s paper, as the criteria to select cases is very similar in the two studies (i.e., obesity and at least one other feature such as DD, ID, congenital anomalies or dysmorphic features). Although whole genome array allows the investigation of known syndromes and abnormalities that have not been described before, yet cost considerations still limit its use in routine clinical application in developing countries, whereas MLPA has been found to be a good option in situations of cost constraints [23,24]. In our experience, the per sample cost of MLPA to CMA is in the ratio of 1:5. Moreover, MLPA does not require validation of copy number changes detected by more than one probe, thus eliminating costs with additional tests for establishing the diagnosis. In addition, the probe set as we have developed it detects only known aberrations so that the clinical interpretation of the data is straightforward.

We diagnosed nine of patients with clinically well-defined microdeletion syndromes complicated by obesity. Among these, four cases had 2q37 deletions ranging from 2.2 to 6.2 Mb. In all patients, except one, the deletions involved *HDAC4* (*histone deacetylase 4*), the primary causative gene for BDMR syndrome [3], and that could be responsible for obesity (seen in >40% of patients [25]). The protein encoded by this gene is involved in histone acetylation and chromatin remodeling. Consistent with a role of *HDAC4* in the development of obesity, deletion in or mutation of *HDAC4* was reported as resulting in reduced expression of the *retinoic acid induced 1* (*RAI1*) gene, whose haploinsufficiency leads to obesity in SMS [3]. Moreover, HDAC4 was shown to be downregulated in obese compared with lean subjects, and then induced by physical activity [26]. Of note, the only two patients with *HDAC4* point mutations reported to date were detected in BDMR patients who were obese [3]. Other two genes, *CAPN10* and *HDLBP*, that are located distal to *HDAC4*, were also associated with obesity [27] and deleted for patient 5 carrying a 2q37 deletion that is distal to and did not include *HDAC4*. Another obese patient with a terminal deletion distal to *HDAC4* was reported by Williams *et al*. [3]. These results may suggest one more distal locus at 2q37.3 critical for obesity.

A relatively small 9q34.3 deletion including only partially the *euchromatic histone methyltransferase 1* (*EHMT1*) gene, in which deletion (seen in >85% of cases) or mutation (rarely) causes KS, was found in one patient. Recent reports support previous observations that obesity is present in a higher frequency in KS [28-30]. In the latest study of 83 KS patients with BMI data, including both patients with deletions and point mutations of *EHMT1*, Williemsem *et al*. [30] showed that ~30-40% of the KS patients were overweight (BMI >25), and observed that this feature was more frequent among patients with a mutation than in patients with a deletion (42 vs. 28%, respectively). In a recent study by Ohno *et al*. [31], it was demonstrated that deficiency of EHMT1 in brown fat cells leads to obesity and insulin resistance. This reinforces the notion that haploinsufficiency of *EHMT1* is causative for obesity. Four patients carried a 17p11.2 deletion, including *RAI1,* the major gene for the phenotypic features of SMS. Obesity has been reported in >50% of SMS patients with either deletion or mutation of the *RAI1* [32,33]. Supporting the fact that *RAI1* is involved in the obesity phenotype, *Bdnf*, a gene associated with obesity and hyperphagia, was found downregulated in the hypothalamus of the mouse model for *Rai1* haploinsufficiency [34]. Furthermore, it was found in the same study that human *RAI1* directly regulates the expression of *BDNF*.

Six patients were diagnosed with chromosome 22q11.2 abnormalities at both proximal and distal intervals. The 22q11.2 deletion spanning the four proximal LCR22s A to D within the DiGeorge (DG) and velo-cardio-facial syndrome (VCFS) region was found in three patients and the reciprocal duplication was found in another. Approximately one in every 167 patients with neurocognitive disorders and multiple congenital anomalies has a deletion in the 22q11.2 DG region, while its reciprocal duplication occurs in 1 of every 384 such cases [35,36]. Two distinct studies have reported a high prevalence of obesity in adolescents and adults with DG [37,38]. In one of these studies, it was shown that at adolescence or early adulthood, up to 35% of DG deletion carriers developed obesity [38]; data on the prevalence of obesity in 22q11.2 duplication has not been published.

We have identified two patients with the 22q11.2 ‘distal’ deletion associated with a variable clinical phenotype [39]. The deletions boundaries for the two individuals are within LCR22s E and F, whereas most reported distal 22q11.2 deletions has been shown to extend from LCR22-D to LCR22s E or F [40,41]. Obesity (weight-for-age >90th percentile) was reported in 6 of 22 previously published cases [39-42], and three patients had a postnatal weight between the 75th and 90th percentile [40,41]. In addition, two of the obese patients reported by Fagerberg *et al*. [40] had neurobehavioral problems such as food seeking behaviors and hyperphagia. Thus, our findings further suggest that the 22q11.2 distal deletion may predispose to obesity and overweight.

Additionally, three other patients were found to have recurrent CNVs associated with incomplete penetrance and variable expressivity. A child presenting with an overgrowth phenotype (macrocephaly, growth acceleration, and obesity) had the distal 1q21.1 duplication previously associated with macrocephaly (the reciprocal deletion is associated with microcephaly) and other features of overgrowth [43-45]. This locus includes the brain-specific *HYDIN2* gene and a cluster of *NBPF* genes, which have been proposed as candidates for the abnormal head size [43,46]. The 16p11.2 distal deletion associated with DD and obesity [16] was found in one individual. This deletion was shown to account for 0.5% of severe childhood obese cases [47], and was reported in 46 of 38851 (0.12%) patients with DD in two recent large studies [36,48]. This locus includes the *SH2B adaptor protein 1* (*SH2B1*) gene, which is likely responsible for obesity in these individuals [16]. Another patient was found with the proximal 16p11.2 duplication associated with neurocognitive deficits and known to increase the risk of being underweight [49], whereas the reciprocal deletion cosegregates with severe early-onset obesity [17]. Both the deletion and the duplication were shown to occur in 0.2-0.4% patients submitted for clinical CMA testing [35,36].

As for the remaining probes in the probe set, we were unable to identify copy number imbalances in patients from our cohort. At least two CNVs, of *SIM1* at 6q16 and *BDNF* at 11p14, are well known to include obesity as a phenotype [6,7,14,50,51]. In addition, 2p25.3 deletions spanning *MYT1L* have now been reported in a number of patients with a PWS-like phenotype [9,10,21]. The Imprinted in Prader-Willi (*IPW*) non-coding RNA is located to the critical region containing the functional PWS gene locus, including the *SNORD109A* and *SNORD116* snoRNA cluster [52]. This minimal region was delineated based on the study of rare cases resulting from atypical 15q11q12 microdeletions without methylation abnormalities [52-55]. Extremely rare mutations in *NTRK2*, which encodes a highly specific receptor for BDNF, were found in severely obese children with DD [56,57]. However, it is currently unknown whether or not CNVs of the *NTRK2* gene also can result in a comparable phenotype. None of the probes within new candidate loci for syndromic obesity (i.e., 3p26.3, 4p16.1, 7q22.1, 12q15q21.1, 14q11.2, and 14q12) [21,58; Table 2], were found as copy number variable in any additional patient. Hence, the contribution of these CNVs to obesity still remains uncertain and yet to be demonstrated.

**Table 2:** **Genomic regions and genes in the MLPA probe set**

**Regions**	**Gene(s)**	**Evidence**	**Refs**
1q21.1	*PRKAB2*	Recurrent deletions and duplications at 1q21.1 are susceptibility factors for a variety of neurodevelopmental phenotypes. In one study, 6 out of 7 adults with 1q21.1 duplications had obesity or overweight and in 2 of 6 children with data, weight was above the 90th percentile. In another study, four patients were described with obesity and 1q21.1 deletions. Obesity was reported in four patients from DECIPHER (249137, 289048, 268066, and 249571) with duplication and in another (DECIPHER 290856) with deletion.	[43-45]
	*ACP6*		
2p25.3	*ACP1*	Deletions of 2pter are rare, and have often been associated with a PWS-like phenotype. The genes *ACP1*, *TMEM18*, and/or *MYT1L* were proposed as obesity candidates.	[9,10,21]
	*TPO*		
	*MYT1L*		
2q37.3	*HDAC4*	Deletions of the chromosome region 2q37 or mutation in the *HDAC4* gene cause BDMR syndrome (obesity is seen in >40% of patients).	[3,25]
	*GPR35*		
3p26.3	*CNTN6*	One patient described with syndromic obesity presenting with a 3pter deletion including only *CNTN6* and *CHL1* genes, both encoding neuronal adhesion molecules. Another patient with obesity reported in DECIPHER (249965) harboring an overlapping deletion in band 3p26.3.	[21]
	*CHL1*		
4p16.1	*ACOX3*	Williams *et al*. reported on a patient with a typical SMS phenotype showing obesity (SMS336) presenting with dup (4)(p16.1).	[58]
	*CPZ*		
6q16.3	*SIM1*	Obese patients presenting with a PWS-like phenotype and 6q16 deletions including *SIM1*. Obesity has been reported in *Sim1* haploinsufficient mice and in a patient with a balanced translocation disrupting *SIM1*. SIM1 is a basic helix-loop-helix transcription factor involved in the development and function of the paraventricular nucleus of the hypothalamus.	[6,21,50]
7q22.1	*RELN*	Two reports of 7q22 deletions in a patient presenting with syndromic obesity, and in another showing overgrowth and obesity.	[21,59]
9q21.33	*NTRK2*	A heterozygous *de novo* mutation in *NTRK2* was found in a child presenting with severe obesity, hyperphagia, and DD. NTRK2 is a highly specific receptor for BDNF, which makes its position within the leptin-melanocortin pathway evident.	[56,57]
9q34.3	*EHMT1*	*EHMT1* deletion (seen in >85% of cases) or mutation (rarely) causes KS. Obesity is present in a higher frequency in KS (~30-40%), and is more prevalent among patients with *EHMT1* mutation than in patients with deletions (42 vs. 28%, respectively).	[30]
11p14.1	*BDNF*	Deletions extending the *BDNF* locus are associated with risk of obesity in a subgroup of patients with WAGR. Deletions outside of the WAGR region but spanning *BDNF* were reported in four patients with DD, behavioral problems, and obesity. Disruption of *BDNF* expression was associated with hyperphagia, obesity, and cognitive impairment in one published patient	[7,14,51]
	*MPPED2*		
12q15q21.1	*PTPRB*	Identical twins with deletion 12q15q21.1 presenting with syndromic obesity.	[21]
	*RAB21*		
	*TPH2*		
14q11.2	*CHD8*	Only one patient described with syndromic obesity presenting with a 14q11.2 microduplication encompassing *SUPT16H* and *CHD8*, highly expressed in adult and fetal brain, *RAB2B*, and two small nucleolar RNA (snoRNAs) [21].	[21]
	*RAB2B*		
14q12	*PRKD1*	Only one patient described with syndromic obesity presenting with a 14q12 microdeletion including only *PRKD1* and a microRNA (*MIR548AI*) gene.	[21]
15q11.2	*IPW*	*IPW* is located to the critical region containing the functional PWS gene locus, and was found deleted in patients with atypical 15q11.2 deletions presenting the major features of PWS but normal methylation analysis.	[52-55]
16p11.2	*CDIPT*	The proximal 600-kb recurrent deletion within 16p11.2 confers susceptibility to autism and often cosegregates with early-onset obesity and neurodevelopmental disorders. The distal recurrent *SH2B1*-containing deletion within 16p11 was shown to account for 0.5% of severe childhood obese cases often co-occurring with DD. *SH2B1* is involved in leptin and insulin signaling and is a solid candidate for obesity.	[16,17]
	*MAPK3*		
	*SH2B*		
17p11.2	*RAI1*	Deletions of the chromosome region 17p11.2 or mutation in the *RAI1* gene cause SMS. Obesity and hypercholesterolemia are phenotypes of SMS. *RAI1* encodes a transcriptional regulator that directly regulates the expression of *BDNF*, a gene associated with obesity and hyperphagia. *Bdnf* was found downregulated in the hypothalamus of the mouse model for *Rai1* haploinsufficiency.	[32-34]
22q11.2	*CRKL*	Obesity in patients from literature with deletions at both proximal and distal chromosome 22q11.2 intervals, and in patients from DECIPHER (2184, 2695, 248709, 250255, and 250888).	[37-42,60]
	*MAPK1*		
	*RAB36*		

## Conclusions

Our study demonstrates the utility of an MLPA-based first line screening test in the evaluation of the genetic etiology of syndromic obesity in 338 patients. The overall detection rate with the synthetic MLPA probe set was about 5.3% (18 out of 338). As compared to chromosomal microarrays, it is an efficient, rapid, less labour intensive and cost-effective alternative for interrogating the copy number status at multiple loci that are known to cause this phenotype. Application of CMA testing to as yet undiagnosed individuals will uncover new loci responsible for the patients’ phenotype, which would otherwise remain undetected based solely on the MLPA evaluation. These could eventually become new microdeletion/duplication syndromes associated with syndromic obesity. Our results also suggest that obesity could likely be a feature of the 22q11.2 distal deletion syndrome. Finally, our experience leads us to suggest that incorporating an MLPA-based first line screening test targeting various loci in which altered dosage is known to result in obesity as a phenotype (such as 1p36, 2p25, 2q37, 6q16, 9q34, 11p14, 16p11.2, and 17p11.2), could provide an effective alternative diagnostic approach to chromosomal microarrays for syndromic obesity, especially in clinical settings where CMA is not available.

## Methods

### Patients

Three hundred and thirty-eight nonrelated individuals with a prior negative methylation test for PWS were included in this study. The study protocol was reviewed and approved by the Human Research Ethics Committee at the Institute of Biosciences, University of São Paulo (CEP/IB/021/2004). Parents or guardians also provided written informed consent. All the patients had a general diagnosis of DD/ID along with obesity or overweight. However, this cohort also includes other phenotypic findings including, but not restricted to, congenital malformations, hypotonia and feeding difficulties, behavioral issues, autism spectrum disorders, hyperphagia, hearing impairment, epilepsy, and dysmorphic features. Part of this cohort had previously been discarded for microdeletions of chromosome 1p36 by the syndrome-specific SALSA MLPA kit (P147, MRC Holland, Amsterdam, The Netherlands) [61]. All DNA samples were obtained from peripheral blood using the Autopure LS® (Gentra Systems, Inc., Minneapolis, MN).

### Multiplex-ligation dependent probe amplification (MLPA)

#### Synthetic MLPA probe set

The probe set includes 31 MLPA probes for the detection of copy number imbalances involving the following chromosomal regions (genes): 1q21.1 (*PRKAB2*, *ACP6*), 2p25.3 (*ACP1*, *TPO*, *MYT1L*), 2q37 (*HDAC4*, *GPR35*), 3p26.3 (*CNTN6*, *CHL1*), 4p16.1 (*ACOX3*, *CPZ*), 6q16 (*SIM1*), 7q22.1 (*RELN*), 9q21.33 (*NTRK2*), 9q34 (*EHMT1*), 11p14 (*BDNF*, *MPPED2*), 12q15q21 (*PTPRB*, *RAB21*, *TPH2*), 14q11.2 (*CHD8*, *RAB2B*), 14q12 (*PRKD1*), 15q11.2 (*IPW*), 16p11.2 (*SH2B1*, *CDIPT*, *MAPK3*), 17p11.2 (*RAI1*) and 22q11.2 (*CRKL*, *MAPK1*, *RAB36*) (Table 2). Up to three probes were designed preferably in coding regions of specific genes within the regions of interest. The probes were designed online using the publicly available MAPD software [62]. Individual oligonucleotide probes (size range 100–168 nt) were synthesized by IDT® (Integrated DNA Technologies, Belgium) and added to the SALSA® MLPA® P200 and P300 Human DNA reference kits (Table 3). The MLPA reactions were performed on 100–250 ng genomic DNA samples following the MRC-Holland protocol [63]. MLPA products were size-separated by capillary electrophoresis on the 3730 Genetic Analyser (Applied Biosystems, UK) and interpreted with the GeneMarker (v1.95) software (SoftGenetics, LLC. State College, PA, USA) using the “population normalization” method. Peak ratios between 0.75 and 1.25 were considered normal (i.e. two copies). Figure 2 shows the MLPA profiles for the two testing panels in control samples of different sex. These panels were validated by the analysis of DNA of patients with known CNVs (data not shown). When available, blood samples were obtained from patients’ parents, and CNV inheritance was investigated.

**Table 3:** **The MLPA probe sequences**

**Probes**	**5’-LPO (Left probe oligonucleotides)**	**3’-RPO (Right probe oligonucleotides)**	**Genomic position (GRCh37/hg19)**	**Length (nt)**
**Added to the SALSA® MLPA® P200 Human DNA reference kit**				
ACP1	TAAGAAATCATGGCATTCACACAGCCCAT	AAAGCAAGACAGGTAGACAAGCTCTTGTT	chr2 + 272265-272322	100
TPO	GAACGAGGAGCTGACGGAAAGGCTCTTTGTG	CTGTCCAATTCCAGCACCTTGGATCTGGCGT	chr2 + 1491663-1491724	104
MYT1L	TCTGCATGCTGCCCGGAGTTGTTGTTAAACATG	AGTCTGTGTATTCAAGGCTAGTTTCCTGGGGCG	chr2 - 2329482-2329547	108
CNTN6	GCATGGACCTTCAATGATAACCCCTTATACGTCCA	AGAGGACAATAGGCGATTTGTATCTCAAGAGACGG	chr3 + 1337296-1337365	112
ACOX3	GGTGGCCAGAGTTTTCTGTGAACAAACCTGTCATAGG	AAGTCTGAAATCGAAGCTCTAGTGGGACTGGCACACA	chr4 - 8368673-8368746	116
CPZ	TCCACCCCATGATGATGGACAGGTCGGAGAATAGGTGTG	GAGGCAATTTCCTGAAGAGGGGGAGCATCATCAACGGGG	chr4 + 8613788-8613865	120
RELN	TCTGCGGGTCATATTCATACCTTCTGATGAAGTTGTACAAC	ACCAGCAACATTATAATGGCCCTGTAGCTCTGAATGCTATT	chr7 - 103112316-103112397	124
CHD8	GCCTTCTTGCAGGAAGTATATAATGTGGGCATCCATGGTCCCT	TCTTGGTCATTGCCCCACTGTCCACAATTACTAACTGGGAGCG	chr14 - 21876574-21876659	128
RAB2B	GAGTGTGCTTTCTCTTTCAGGTGTGGGGAAGTCATGTCTCCTCCT	GCAGTTTACAGATAAGCGGTTCCAGCCTGTCCACGACCTCACAAT	chr14 - 21944689-21944778	132
CRKL	TAGTGATAATAGAGAAGCCTGAAGAACAGTGGTGGAGTGCCCGGAAC	AAGGATGGCCGGGTTGGGATGATTCCTGTCCCTTATGTCGAAAAGCT	chr22 + 21288204-21288297	136
MAPK1	GTCCTTCGTTATGTTCCCCAGATGTCTTCCAGATTTGCTCTGCATGTGG	TAACTTGTGTTAGGGCTGTGAGCTGTTCCTCGAGTTGAATGGGGATG	chr22 - 22114451-22114546	140
RAB36	CACAGGTTTTGCAAGAATGTTTTTGATCGAGACTACAAGGCCACCATTGGG	GTGGACTTTGAAATTGAGCGCTTTGAGATTGCTGGGATTCCCTATAGCCTC	chr22 + 23495215-23495316	144
PTPRB	GGGAATGTGGAACGATACCGGCTGATGCTAATGGATAAAGGGATCCT	AGTTCATGGCGGTGTTGTGGACAAACATGCTACTTCCTATGCTTTTCACGGGC	chr12 - 70988331-70988430	148
TPH2	CAGGGTGGAGTATACTGAAGAAGAAACTAAAACTTGGGGTGTTGTATTCCGGGAG	CTCTCCAAACTCTATCCCACTCATGCTTGCCGAGAGTATTTGAAAAACTTCCCTC	chr12 + 72366314-72366423	152
RAB21	GAGCAGAGGAAGAGATCCCAGATAGTAGCCAGTTAACCAAGACTCATTCATATAGCA	CGTAGTTTATGTTCCTGAGGCAGCACTTTTAGATCCTTTGTGAGCAAGTTCTATTTG	chr12 + 72180755-72180868	156
CHL1	GGTGATGTTGTCTTCCCCAGGGAAATCAGTTTTACCAACCTTCAACCAAATCATACTGC	TGTGTACCAGTGTGAAGCCTCAAATGTCCATGGAACTATCCTTGCCAATGCCAATATTG	chr3 + 401981-402098	160
PRKD1	GCCACCTTTGAAGACTTTCAGATTCGTCCCCACGCTCTCTTTGTTCATTCATACAGAGCTC	CAGCTTTCTGTGATCACTGTGGAGAAATGCTGTGGGGGCTGGTACGTCAAGGTCTTAAATG	chr14 - 30135288-30135409	164
IPW	TTGCCCATTTATCTGTACCGCCATCTTGCGCATATGCTGTACTCTCATCTGTGACTGGCTCCA	TTTTTGTTCTGTGGATTTGTGTGTCTCTTCTTCTGCCTCCTGTCTCGTGTCTGCTCGTTGGAA	chr15 + 25365689-25365814	168
**Added to the SALSA® MLPA® P300 Human DNA reference kit**				
PRKAB2	TTCTTGCTGTCTTCTACCAGGGGCTGCTG	ACTCCAGTTACCCATGGAATGCAGGACCT	chr1 - 146627622-146627679	100
ACP6	TCTAGCTGGTGGTCCGAAACCATATTCTCCT	TACGACTCTCAATACCATGAGACCACCCTGA	chr1 - 147131764-147131825	104
HDAC4	GCCGTGGCCACCATTCACCTCTGTAATTTAATCCGT	TTCTCTTGGATTGTCTGGACGTGCCCGATGGTTCTT	chr2 - 239970066-239970137	114
GPR35	TGTACATAACCAGCAAGCTCTCAGATGCCAACTGCTGC	CTGGACGCCATCTGCTACTACTACATGGCCAAGGAGTT	chr2 + 241570142-241570217	118
SIM1	GAAGAGAACAGATTACAGCTAAGGAAAGCCCCCTCAGACC	AACTGGCTTCCATTAATGGGGCTGGGAAAAAACACTCCCT	chr6 - 100838727-100838806	122
NTRK2	CTAGTGTTGCAGTATAGCTTTGGCATGTTCATGAGTGAGCACCCAG	AATGTGTTGAACCAACCCCCACCCCTAACTACTGACTATGACTGCA	chr9 + 87430124 -87430215	134
EHMT1	CCTCTAACTGACGTTTCTTTTCGAGGAAGTGGCTTGGTGGGTGCAGCC	CCCGCCGGTTCCGTTGACGCTGGCACCTTCTGTTGATTTTTTAAGCCA	chr9 + 140730014-140730109	138
BDNF	CCTCATTGAGCTCGCTGAAGTTGGCTTCCTAGCGGTGTAGGCTGGAATAG	ACTCTTGGCAAGCTCCGGGTTGGTATACTGGGTTAACTTTGGGAAATGCA	chr11 - 27741944 -27742043	142
MPPED2	GTTATGGCATCATGACCGACGGTTACACAACGTACATCAATGCCTCGACGTGTAC	AGTCAGCTTTCAACCGACCAACCCTCCAATTATATTTGACCTTCCAAACCCACAG	chr11 - 30433024-30433133	152
SH2B1	GATTGGTTTGCACTTCTTGGCTGGGTTCCCCCGTGCTCCATGACTCCTGCATCTCCT	GATTGTTTCTCGTTGGTTTGGAGTTGTCCCTGCGGTTGGAGCCATCTGAGCTTGTAG	chr16 + 28875745-28875858	156
CDIPT	TAGGAGGTCCCAGTCTCACGCCTTCCTCATGTGTTGTTCTACCTGCTGGGATGGGGGTC	AGCCTCTCTTTGGTGACGTCACGTTCTCTGGGATCCTGAGGACCCGGGCCTCAAATCAG	chr16 - 29870318-29870435	160
MAPK3	GACTCGCGTGGCCATCAAGAAGATCAGCCCCTTCGAACATCAGACCTACTGCCAGCGCACG	CTCCGGGAGATCCAGATCCTGCTGCGCTTCCGCCATGAGAATGTCATCGGCATCCGAGACA	chr16 - 30133182-30133303	164
RAI1	TCGCTACGCCTGACCCCAAAAAGACAACTGGTCCTCTCTCCTTTGGTACCAAGCCCACCCTTG	GGGTTCCTGCTCCAGACCCCACTACAGCAGCTTTTGACTGTTTCCCGGACACAACCGCTGCCA	chr17 + 17698343-17698468	168

LPO: starts with GGGTTCCCTAAGGGTTGGA (forward primer binding sequence).

RPO: ends with TCTAGATTGGATCTTGCTGGCAC (reverse primer binding sequence).

#### Follow-up MLPA kits

When an alteration was found within the 17p11.2 (SMS) region or in 22q11.2 within the DG/VCFS region, confirmatory testing was performed with commercial MLPA kits (MR-1 MLPA kit P064-B2 and DG/VCFS MLPA kit P023-B, respectively).

### Chromosomal microarray analysis (CMA)

The alterations in 10 patients were verified using at least one array platform. The arrays used in this study are the Affymetrix 500 K SNP-array (Affymetrix, Santa Clara, CA, USA), the CytoSure ISCA 180 K oligoarray-CGH (Oxford Gene Technology, Oxford, UK), and a 60 K custom-designed 60-mer oligoarray (Agilent Technologies, Palo Alto, CA, USA). SNP-array testing was performed as previously described [21]. Oligoarrays were hybridized according to the manufacturer’s’ instructions. Hybridizations were performed in duplicates with dye-reversal method. Scanned images were processed using Agilent Feature Extraction software and analyzed with Genomic Workbench software (both from Agilent Technologies) applying the statistical algorithm ADM-2 with a sensitivity threshold of 6.7. Duplication or deletion was considered when the log_2_ ratio of the Cy3/Cy5 intensities of a given region encompassing at least three probes was >0.3 or - < 0.3, respectively. Genomic coordinates were converted to UCSC genome browser build February 2009 (GRCh37/hg19).

## Abbreviations

DD: Developmental delay
ID: Intellectual disability
MLPA: Multiplex ligation-dependent probe amplification
CNV: Copy number variant
CMA: Chromosomal microarray analysis
BMI: Body mass index
PWS: Prader-Willi syndrome
BDMR: Brachydactyly-mental retardation
KS: Kleefstra syndrome
WAGR: Wilms tumor, aniridia, genitourinary anomalies, and mental retardation
SMS: Smith-Magenis syndrome
DG: DiGeorge
VCFS: Velo-cardio-facial syndrome
SD: Segmental duplication
LCR: Low-copy repeat
